# High serum phosphate and triglyceride levels in smoking women and men with CVD risk and type 2 diabetes

**DOI:** 10.1186/1758-5996-6-39

**Published:** 2014-03-17

**Authors:** Lena M Håglin, Birgitta Törnkvist, Lennart O Bäckman

**Affiliations:** 1 Department of Public Health and Clinical Medicine, Family Medicine, Umeå University, UMEÅ SE-901 87, Sweden; 2 Animal Health Care, Hällnäs 4, Vännäs SE- 911 94, Sweden

**Keywords:** Glucose intolerance, Obesity, Risk factors, Gender, Phosphate, Smoking

## Abstract

**Background:**

Both low and high serum phosphate levels may be associated with morbidity and mortality from cardiovascular disease. As smoking increases risk for type 2 diabetes (as shown by dyslipidemia and hyperglycemia), we wanted to study whether smoking and type 2 diabetes were associated with serum phosphate and triglyceride levels independently from other CVD risk factors.

**Methods:**

Upon admittance to the Vindeln Health Education Centre (VHE-centre) for a four-week comprehensive lifestyle intervention, the participants (1408 women and 1096 men) completed a questionnaire that included their smoking habits – current smoker or non-smoker. We used multiple linear regression analyses to investigate the association between smoking and other CVD risk factors with S-P and S-TG levels.

**Results:**

In the non-type 2 diabetes populations, the smokers, compared to the non-smokers, had higher S-P and higher serum triglycerides (S-TG). In women, serum-TG in smokers with type 2 diabetes was higher than in smokers with non-type 2 diabetes. Non-type 2 diabetes patients exhibited an inverse relation between S-Glucose (S-Glu) and S-P and a positive association with S-TG. For men only, an association was seen between age (-) and S-Crea (-) and S-P. For women only, an association was seen between BMI (-) and S-Cholesterol (+) (S-Chol) and S-P.

**Conclusions:**

Compared to non-smokers, smoking women with non-type 2 diabetes and smoking men with type 2 diabetes had a higher level of S-P and S-TG. The association between smoking and S-P and S-TG levels still existed after adjusting for age and CVD risk factors in the multiple linear regression analyses.

**Trial registration:**

The study has been registered as a sub-study to the Lifestyle Intervention Trial no. ISRCTN79355192.

## Background

Optimal serum phosphate (S-P) is an important condition for effective glucose metabolism. In glucose intolerance, there is a negative association between S-Glucose (S-Glu) and S-P [1-4]. The low S-P in connection with obesity indicates an association with biomarkers for metabolic syndrome and cardiovascular disease (CVD) risk factors [4-6]; however, high levels of phosphate, although within normal limits, can be an even stronger marker for CVD risk with or without renal disease [7-10]. Thus, defining normal S-P levels may be important in understanding early metabolic disturbances. Reducing high S-P in patients with Chronic Kidney Disease (CKD) is an important therapy as well as a possible preventive strategy in other patients and individuals with high S-P levels [10]. Therefore, phosphate binders could be used in non-uremic vascular disease to reduce S-P levels [11]. The CVD risk of high levels of phosphate with respect to CKD relative to the risk of low S-P due to obesity has been described as a double-edge sword [4,8,12]. If this U-shaped relationship between S-P and CVD risk is true, the possible links, causes, and mechanisms need more study. Defining the level of S-P that influences CVD risk, either low or high, needs to be determined and used as the normal reference interval in future investigations. As smoking increases risk for type 2 diabetes (as shown by dyslipidemia and hyperglycemia), we wanted to study whether smoking and type 2 diabetes were associated with serum triglycerides and phosphate levels independently from other CVD risk factors.

## Subjects and methods

### Study population

The study population included 2504 patients (1408 women and 1096 men) admitted to the Vindeln Health Education Centre (VHE-centre) between 1984 and 1996 in groups of 30 for a four-week residential comprehensive treatment. The patients had a clinical and biochemical assessed diagnosis and were referred from primary care and hospitals in the county (64%, hypertension; 20%, type 2 diabetes; and 55%, BMI >30 (kg/m^2^). The diagnosis was re-assessed and confirmed by the physician at the health care centre. In addition to the main diagnosis, about half of the patients had a second subsidiary diagnosis, and the prevalence of multiple risk factors resembling metabolic syndrome was high. A second subsidiary diagnosis was never type 2 diabetes, chronic kidney disease (CKD), or coronary heart disease (CHD). At admittance, the participants completed a lifestyle questionnaire that included questions about smoking habits – smoker or non-smoker – at time of admittance. Former smokers were considered non-smokers. The baseline characteristics for former smokers and smokers were significantly different in six out of 10 variables, including S-P and S-TG, indicating reversed level of risk. The personal characteristics of the patient population, separated into women and men, are described in Table 1. A recent published study has described the lifestyle intervention program used by patients in this study and the changes in the metabolic profile for the non-type 2 diabetes and type 2 diabetes patients included in the present study [13].

**Table 1:** Personal characteristics and CVD risk variables in mean (sd) for all patients and for women and men separately

**Variable**	**All participants N = 2504**	**Women N = 1408**	**Men N = 1096**	**Women vs. Men P-value**
Age, yrs	50.4 (10.1)	50.1 (10.7)	50.8 (9.4)	0.073
BMI, kg/m^2^	31.2 (5.4)	31.5 (5.7)	30.7 (4.9)	0.000
Smokers, %	20.9	19.8	22.3	0.136
Type 2 diabetes, %	19.8	17.5	22.7	0.000
SBP, mmHg	147 (19)	145 (19)	148 (19)	0.000
DBP, mmHg	88 (11)	87 (12)	90 (11)	0.000
S-Glu, mmol/l	6.54 (3.23)	6.33 (3.02)	6.80 (3.48)	0.000
S-Chol, mmol/l	6.66 (1.41)	6.63 (1.39)	6.70 (1.44)	0.186
S-TG, mmol/l	2.46 (1.85)	2.18 (1.49)	2.81 (2.18)	0.000
S-Urate, mmol/l	340 (82)	316 (75)	371 (81)	0.000
S- Crea, mmol/l	82.4 (15.2)	76.2 (12.7)	90.2 (14.5)	0.000
S-Ca, mmol/l	2.34 (0.10)	2.35 (0.10)	2.34 (0.09)	0.000
S-Mg, mmol/l	0.83 (0.13)	0.83 (0.13)	0.84 (0.13)	0.019
S-P, mmol/l	1.02 (0.21)	1.05 (0.21)	0.98 (0.20)	0.000

P-value for t-test of mean difference between men and women.

### The life style intervention program

A problem-based learning perspective was established during the first four weeks, and 114 full-time hours were dedicated to food preferences, selections, physical exercise, and stress management. The activities were repeated during a four-day revisit to the centre after one or five years to establish functioning groups as a source to achieve changes and as a start for individual targets. The main focus was to combat risk of future cardiovascular disease (CVD). One outcome was an individual in-home programme. After the residential period, the patients were expected to practice their in-home programme in their habitual environment. During these revisits, the patients and staff revised the in-home programme according to the patients’ experiences during the previous year and their health status.

The Ethical Committee of North Sweden at the University of Umeå (Umeå, Sweden) approved the protocol on November 22, 2006 (Dnr 05-177 M).

### Physical and biochemical variables

On the second day after being admitted to the VHE centre, systolic blood pressure (SBP; mmHg), body weight (kg), height (cm), and body mass index (BMI; kg/m^2^) were recorded. For every patient, a trained nurse measured blood pressure using a semi-automatic machine. Blood was analysed on the serum sample according to the standard routines developed at the Department of Clinical Chemistry, University Hospital, Umeå, Sweden.

Serum glucose (S-Glu) was determined using a hexokinase method (Boehringer Mannheim Diagnostica, Mannheim, Germany) on either a Hitachi 717 or a Hitachi 911 analyser. Serum cholesterol (S-Chol) was determined using an enzymatic method (Boehringer Mannheim Diagnostica, Mannheim, Germany) on either a Hitachi 705 or Hitachi 717 analyser. Serum triglycerides (S-TG) were determined using enzymatic methods (Boehringer Mannheim Diagnostica, Mannheim, Germany) on a Hitachi 717 analyser. Serum urate (S-Urate) was determined using an enzymatic Uricase method (Boehringer Mannheim Diagnostica, Mannheim, Germany) on a Hitachi 705 or Hitachi 717. Serum calcium (S-Ca) was determined using a complexometric method from Boehringer Mannheim on SMA II (Technicon) and on a Hitachi 717 analyser (after 1989). Until 1989, serum magnesium (S-Mg) was determined using the atom absorption technique; after 1989, serum magnesium was determined using a colorimetrically-complex method with reagents from Boehring Mannheim on a Hitachi 717 analyser. Serum phosphate (S-P) was determined using an ammonium-molybdate method (Boehringer Mannheim Diagnostica, Mannheim, Germany) on a Boehringer Mannheim (SMA II Technicon) and on a Hitachi 717 analyser (after 1989). Serum creatinin was analysed by Jaffé method with dialysis on a SMAII (Technicon) before 1989; thereafter and up to 1996, serum creatinin was analysed by an enzymatic creatinin PAP (BM) method on a Hitachi 717 analyser (BM).

### Statistical analysis

**Figure 1:**
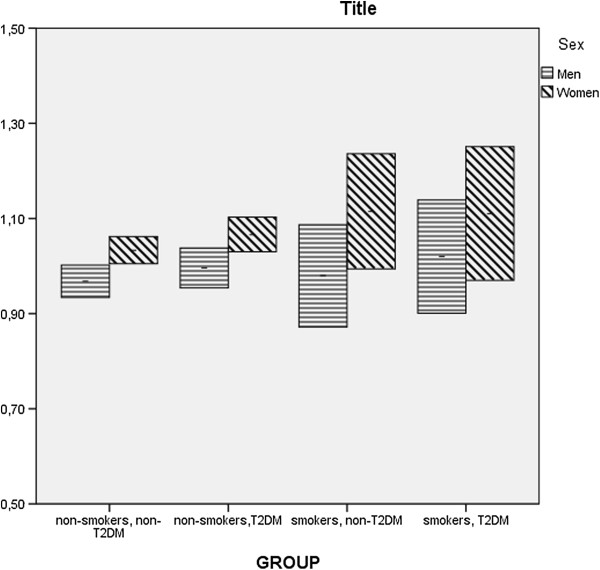
Marginal means of Serum-Phosphate (mmol/l) with upper and lower confidence limits (95%) for the four groups and for men and women.

Student’s t-test was used to analyse differences in personal characteristics and CVD risk variables, and the Chi-square test was used to analyse differences between proportions. All p-values were based on two-sided tests. Interactions with type 2 diabetes were tested using backwards elimination and were included if significant. To study the relationship between CVD risk factors and S-P and S-TG values, we used multiple linear regression analyses [14]. The SAS program pack (version 9.3) was used for the computerized analyses and the SPSS (IBM version 20) for data in Figure 1.

## Results

### Women compared with men

All personal characteristics and CVD risk variables included were different for women and men, except for prevalence of smokers (%), age, and S-Chol (Table 1). Compared to the men, the women had a higher BMI, S-P, and S-Ca and a lower SBP, DBP, S-TG, S-Crea, S-Urate, S-Mg, and S-Glu. The prevalence of type 2 diabetes was lower for women than for men (Table 1). Therefore, we stratified for sex in the comparisons between smokers and non-smokers and non-diabetes and type 2 diabetes patients.

### Smokers compared with non-smokers

Table 2 (women) and Table 3 (men) provide the metabolic profile in mean (sd) for smokers and non-smokers for the diagnosis non-type 2 diabetes and type 2 diabetes at time of admittance. In all four sub-groups, smokers were younger and had higher S-TG levels than non-smokers.

**Table 2:** Mean (sd) for baseline characteristics in women, smokers vs. non-smokers for non-type 2 diabetes and type 2 diabetes

	**Non- type 2 diabetes**			**Type 2 diabetes**				
**Variable**	**Smokers N = 234**	**Non- smokers N = 927**	**P-value**	**Smokers N = 45**	**Non- smokers N = 202**	**P-value**	**A Smokers**	**B Non-smokers**
Age, yrs	46.1 (11.0)	50.86 (10.1)	0.000	47.6 (11.1)	51.6 (11.7)	0.037	0.4063	0.401
BMI, kg/m^2^	31.2 (5.8)	31.5 (5.7)	0.515	30.8 (6.5)	32.4 (5.3)	0.083	0.7059	0.030
SBP, mmHg	141 (18.9)	146 (19.0)	0.000	143 (17.0)	146.7 (19.0)	0.198	0.625	0.728
DBP, mmHg	85 (12)	88 (12)	0.001	85 (12)	87 (11)	0.332	0.942	0.254
S-Glu, mmol/l^2)^	5.45 (1.35)	5.36 (1.07)	0.370	12.40 (5.08)	10.50 (4.40)	0.015	0.000	0.000
S-Chol, mmol/l	6.58 (1.30)	6.65 (1.39)	0.442	7.09 (1.80)	6.48 (1.34)	0.037	0.077	0.100
S-TG, mmol/l	2.22 (1.19)	2.01 (1.14)	0.015	3.55 (2.63)	2.60 (2.40)	0.018	0.002	0.001
S-Urate, mmol/l	316 (72)	320 (74)	0.387	297 (79)	302 (81)	0.727	0.123	0.002
S-Crea, mmol/l	74.4 (11.4)	77.4 (11.7)	0.000	73.5 (24.2)	75.1 (12.8)	0.663	0.891	0.014
S-Ca, mmol/l	2.34 (0.08)	2.35 (0.1)	0.227	2.38 (0.09)	2.36 (0.11)	0.286	0.004	0.080
S-Mg, mmol/l	0.84 (0.12)	0.84 (0.12)	0.854	0.80 (0.11)	0.78 (0.13)	0.191	0.069	0.001
S-P, mmol/l	1.08 (0.20)	1.05 (0.21)	0.032	1.07 (0.16)	1.05 (0.20)	0.577	0.764	0.654

A = comparing smokers with non-type 2 diabetes (n = 234) and smokers with type 2 diabetes (n = 45).

B = comparing non-smokers with non-type 2 diabetes (n = 927) and non-smokers with type 2 diabetes (n = 202).

P-values for t-test of mean differences between smokers and non-smokers and non-diabetes and type 2 diabetes.

**Table 3:** Mean (sd) for baseline characteristics in men, smokers vs. non-smokers for non-type 2 diabetes and type 2 diabetes

	**Non- type 2 diabetes**			**Type 2 diabetes**				
**Variable**	**Smokers N = 185**	**Non smokers N = 662**	**P-value**	**Smokers N = 59**	**Non smokers N = 190**	**P-value**	**A Smokers**	**B Non-smokers**
Age, yrs	47.5 (8.5)	50.9 (9.5)	0.000	50.8 (9.8)	53.5 (8.9)	0.044	0.012	0.001
BMI, kg/m^2^	30.5 (4.6)	30.9 (5.0)	0.298	29.4 (4.8)	30.8 (4.9)	0.057	0.119	0.735
SBP, mmHg	143 (18)	150 (19)	0.000	144 (18)	149 (19)	0.072	0.819	0.603
DBP, mmHg	87 (11)	91 (11)	0.000	88 (11)	89 (11)	0.324	0.611	0.066
S-Glu, mmol/l^2)^	5.64 (1.64)	5.57 (1.40)	0.582	11.80 (5.4)	10.70 (4.66)	0.128	0.000	0.000
S-Chol, mmol/l	6.76 (1.54)	6.71 (1.37)	0.691	6.78 (1.49)	6.59 (1.59)	0.436	0.949	0.355
S-TG, mmol/l	3.17 (2.54)	2.61 (1.99)	0.006	3.65 (3.02)	2.91 (2.00)	0.031	0.230	0.062
S-Urate, mmol/l	376 (79)	388 (73)	0.044	316 (91)	327 (83)	0.393	0.000	0.000
S-Crea, mmol/l	86.5 (12.5)	91.5 (13.4)	0.000	85.4 (17.9)	89.0 (16.6)	0.154	0.603	0.050
S-Ca, mmol/l	2.34 (0.09)	2.33 (0.09)	0.565	2.33 (0.09)	2.34 (0.12)	0.390	0.717	0.214
S-Mg, mmol/l	0.85 (0.16)	0.84 (0.13)	0.644	0.85 (0.13)	0.81 (0.11)	0.020	0.915	0.001
S-P, mmol/l	1.01 (0.21)	0.97 (0.21)	0.022	1.02 (0.19)	0.97 (0.16)	0.045	0.694	0.966

A = comparing smokers with non- type 2 diabetes (n = 185) and smokers with type 2 diabetes (n = 59).

B = comparing non smokers with non- type 2 diabetes (n = 662) and non smokers with type 2 diabetes (n = 190).

P-values for t-test of mean differences between smokers and non-smokers and non-diabetes and type 2 diabetes.

For non-type 2 diabetes women and men, smoking was associated with low SBP, DBP, and S-Crea and high S-P levels and high S-TG (Tables 2 and 3, respectively). For type 2 diabetes women, smoking was associated with high S-Glu and high S-Chol (Table 2), whereas smoking type 2 diabetes men had low S-Urate, high S-Mg and S-P (Table 3).

### Smokers compared with smokers

Comparing women who smoke (Table 2, p-value in Column A and Table 4), high S-TG and high S-Glu in type 2 diabetes (3.55 vs. 2.60 and 12.40 vs. 10.50, respectively) was not accentuated by smoking when tested in the multiple linear regression. Smoking induced a higher S-Ca in women with type 2 diabetes compared to smoking in non-type 2 diabetes women (2.38 vs. 2.34). This result seems to be an adverse effect from smoking for type 2 diabetes as women with type 2 diabetes who were smokers and women who were non-smokers produced non-significant differences (Table 2).

**Table 4:** **Multiple linear regression analysis with S-TG values as the dependent variable (Standardized parameter estimates and p-value and R**^**2**^**)**

	**Women N = 1408**		**Men N = 1096**	
**Variable**	**Par est**	**p-value**	**Par est**	**p-value**
Age, yr.	-0.012	0.655	-0.013	0.642
BMI, kg/m^2^	0.032	0.185	0.113	0.000
Smoking	0.069	0.003	0.108	0.000
SBP, mmHg	0.009	0.702	-0.032	0.250
S-Glu, mmol/l	0.317	0.000	0.264	0.000
S-Chol, mmol/l	0.288	0.000	0.441	0.000
S-Urate, mmol/l	0.189	0.000	0.133	0.000
S-Crea, mmol/l	-0.034	0.148	-0.015	0.610
S-Ca, mmol/l	0.034	0.156	0.001	0.975
S-Mg, mmol/l	-0.060	0.012	-0.073	0.008
S-P, mmol/l	-0.002	0.941	0.021	0.441
Type 2 diabetes	-0.483	0.000	0.267	0.047
Type 2 diabetes *S-Glu	-0.285	0.017	-0.184	0.135
Type 2 diabetes *S-Chol	0.733	0.000	-0.162	0.180
R^2^	29.4%		25,2%	

Comparing men who smoke, besides the low S-Urate and higher S-Glu (316 vs. 327 and 11.80 vs. 10.70, respectively) S-TG was further accentuated by smoking confirmed in the linear regression model (Table 3, p-value in Column A and Table 4).

### Non-smokers compared with non-smokers

For non-smoking women and men, the difference in S-Mg between non type 2 diabetes and type 2 diabetes was highly significant. Thus, smoking may not be involved in low S-Mg levels in type 2 diabetes. Women with type 2 diabetes had higher BMI, S-Glu and S-TG but lower S-Urate, S-Crea and S-Mg than women with non-type 2 diabetes. Men with type 2 diabetes had higher S-Glu but lower S-Urate, S-Crea and S-Mg.

### Multiple linear regression with S-P as the dependent variable

The level of S-P constitutes the dependent variable in the multiple linear regression analysis and most common risk factors for CVD were included as confounding independent variables in the test of association between smoking and type 2 diabetes and the level of S-P (Table 5). Type 2 diabetes diagnosis interacted with S-Glu in both women and men and with S-Chol in women only, so it was included in the analysis (Table 5).

**Table 5:** **Multiple linear regression analysis with S-P values as the dependent variable (Standardized parameter estimates and p-value and R**^**2**^**)**

	**Women N = 1408**		**Men N = 1096**	
**Variable**	**Par est**	**p-value**	**Par est**	**p-value**
Age, yr.	-0.017	0.569	-0.137	0.000
BMI, kg/m^2^	-0.156	0.000	-0.049	0.123
Smoking	0.053	0.047	0.064	0.032
SBP, mmHg	-0.030	0.278	-0.045	0.132
S-Glu, mmol/l	-0.209	0.008	-0.249	0.002
S-Chol, mmol/l	0.088	0.005	-0.007	0.859
S-TG, mmol/l	-0.002	0.941	0.026	0.441
S-Urate, mmol/l	0.098	0.001	0.051	0.130
S-Crea, mmol/l	0.017	0.540	0.061	0.049
S-Ca, mmol/l	0.091	0.001	0.182	0.000
S-Mg, mmol/l	0.169	0.000	0.168	0.000
Type 2 diabetes	0.158	0.251	0.027	0.854
Type 2 diabetes *S-Glu	0.375	0.006	0.293	0.029
Type 2 diabetes *S-Chol	-0.312	0.013	-0.084	0.525
R^2^	8.7%		11.0%	

Smoking (+), S-Glu (-), S-Ca (+), S-Mg (+), and the interaction between type 2 diabetes and S-Glu (+) was significantly associated with the S-P levels in both women and men. The inverse relationship between S-Glu and S-P existed only for non-type 2 diabetes patients (i.e., the higher S-Glu, the lower S-P levels). The multiple linear regression analysis also revealed that women with type 2 diabetes had higher S-P the lower their S-Chol level, and non- type 2 diabetes women had higher S-P the higher their S-Chol level. For men but not for women, an association was seen for age (-) and S-Crea (+) with the level of S-P.

### Multiple linear regression with S-TG as the dependent variable

The level of S-TG constitutes the dependent variable in the multiple linear regression analysis and most common risk factors for CVD were included as confounding independent variables in the test of association between smoking and type 2 diabetes and the level of S-TG (Table 4). Associations between smoking (+), B-Glu (+), S-Chol (+), S-Urate (+), and S-Mg (-) and S-TG were shown for both women and men. In women only, type 2 diabetes (-) and its interaction with both glucose (-) and cholesterol (+) were strong and significantly associated with S-TG (Table 4). In men only, type 2 diabetes (+) and BMI (+) were associated with S-TG (Table 4).

## Discussion

We found a higher level of S-P and S-TG in smokers compared to non-smokers in non- type 2 diabetes women and men. These associations still existed after adjusting for age and CVD risk factors in the multiple linear regression analysis. No interaction between smoking and type 2 diabetes in the association with S-P levels was revealed while interaction between S-Glu and type 2 diabetes was positively associated with S-P. This indicates an existence of high but normal S-P levels in type 2 diabetes as well (Figure 1). The adverse and combined association between CVD risk with smoking in type 2 diabetes may partly be due to high S-P levels in addition to a high S-TG levels.

An inverse relationship between S-Glu and S-P in non-type 2 diabetes women and men, between age and S-P in men, and BMI and S-P in women indicate the need to adjust for sex, age, and BMI in the assessment of CVD risk factors associated with S-P levels. Age-related differences in S-P between women and men may be due to differences in renal thresholds for phosphate [15]. The comparison of smokers with non-smokers after stratification for sex reveals higher S-P as well as higher S-TG levels and a lower SBP and DBP in both women and men. In a community study, a higher prevalence of smokers was seen in the highest quartile of serum phosphate [9]. Some studies have reported higher but within normal levels of S-P for smokers without a discussion of the importance of S-P with respect to either CVD risk or insulin resistance for smokers [9,16]. The present study highlights associations between some conventional CVD-risk factors and high but normal S-P levels and as such expands our knowledge of the U-shaped risk pattern related to CVD risk, not including patients with kidney disease and hyperphosphatemia. In chronic kidney disease, a pathological level of S-P (hyperphosphatemia) is frequently reported as S-P may interact with calcium, which increases CVD risk [8].

Increased levels of S-TG and S-P in smokers and type 2 diabetes patients compared with non-smokers and non-type 2 diabetes patients indicate a common disturbance of metabolism linked to CVD risk associated with insulin resistance. A high level of glycosylated haemoglobin (HbA1c) in smokers may be an indicator for insulin resistance, but the mechanism behind this is still unclear [17]. In an earlier study, S-P was shown to be a marker for glycaemia control [18].

In light of this study, future research should address the following question: Does the high normal level of S-P signal insulin resistance in smokers and in patients with type 2 diabetes? Factors contributing to a high level of S-P in smokers are largely unknown and sources and mechanisms responsible for an increase in S-P in smokers and the association between level of S-Glu and level of S-P in type 2 diabetes needs further investigation.

There are three possible mechanisms or explanations for a high level of S-P that may be present in smokers and in patients with type 2 diabetes: 1) an increased threshold for reabsorption of phosphate in the kidney tubules can partly be explained by a decrease in the level of parathyroid hormone (PTH) [15,19]; 2) low bone mineral content (BMC) analysis has shown that smokers have greater bone loss than non-smokers perhaps as a result of mobilization of phosphate from bone by either increased resorption or decreased mineralisation revealed [20]; and 3) a decreased cellular uptake of glucose for intracellular energy metabolism (i.e., phosphorylation) may be related to both low oxygen consumption and the level of S-P, described as affinity hypoxia [3].

We believe that the higher S-P may be associated with intracellular depletion of Pi, oxygen, and glucose. A disturbed oxidative phosphorylation may explain lower ATP production in offspring who have parents with type 2 diabetes [21] and this may also explain why recovery from exercise in smokers involves a delay in adenosine triphosphate (ATP) resynthesis [22]. Intracellular phosphate depletion may also underlie hyperinsulinemia [23].

The important findings that type 2 diabetes was associated with S-TG – although in opposite directions in women (-) and men (+) and stronger for women than men (p = 0.000 vs. p = 0.047) – may be associated with the level of other risk factors in the model and, for women, associated with the level of S-Cholesterol. In women only, the interaction between type 2 diabetes and cholesterol and S-TG could explain the opposite directions of associations. Smoking was strongly associated with S-TG after control for the other CVD risk factors resembling metabolic syndrome.

Another important finding that links these two conditions (metabolic disturbance in smokers and in type 2 diabetes) to insulin resistance and CVD risk is dyslipidemia (i.e., high S-TG). We found that high S-TG in type 2 diabetes men but not women was further accentuated by smoking (i.e., higher S-TG in smokers vs. non-smokers). Smokers had higher visceral obesity as shown by a high waist/hip ratio [24]. Thus smoking is associated with dyslipidemia and central fat accumulation [25]. A study on men with and without cardiovascular disease found that S-TG levels were higher in smokers than non-smokers [26]. Smoking and very high levels of TG were associated with myocardial infarction in women with diabetes mellitus [27]. The higher S-TG might indicate (in addition to an increase in S-Glu) that insulin dependent glucose transport is affected and the increased risk of metabolic syndrome with smoking may be associated with high S-TG but not linked to insulin resistance [28]. The higher S-TG in smokers indicates a disturbed metabolism of fat (i.e., disturbed β-oxidation). In addition, the lack of oxygen entering a cell (affinity hypoxia) may negatively affect glucose transport and oxidative phosphorylation [12], eventually contributing to reduced combustion of fat. Nocturnal intermittent hypoxia was associated with increased risk of developing type 2 diabetes, a finding that supports the present hypothesis [29].

The weakness of this study was the cross-sectional design – descriptive data from a non-randomly selected population. Another limitation was the lack of adjustments of alcohol consumption and social differences. In addition, we had no access to information regarding insulin levels or measures for insulin resistance.

## Conclusion

This study reveals that smoking is associated with higher although within normal limits of S-P levels and that S-Glu is negatively associated with S-P in non-type 2 diabetes patients, indicating glucose intolerance. Increased levels of S-TG and S-P in smokers and type 2 diabetes patients compared with non-smokers and non-type 2 diabetes patients indicate a common disturbance of metabolism linked to CVD risk associated with insulin resistance. Future studies on the causal relation between cigarette smoking and risk for insulin resistance should address S-P levels and include the possible effects of smoking cessation on reducing S-P and S-TG levels.

In every study of metabolic syndrome and obesity in relation to CVD risk, associations and influences from smoking need to be considered as smoking independently contributes to both high S-TG and high S-P levels. Although the smoking-S-TG association was stronger than what was found for S-P, this result provides information for future studies on CVD risk and high S-P levels in general. As our study shows, smoking needs to be considered as a confounder if not a causative factor.

### Patient consent

Obtained.

## Competing interests

There are no conflicts of interest.

## Authors’ contributions

The first author LH (corresponding author) designed the study with support from BT, the statistician (PhD). Data files and calculations were performed by LB (MD). All three authors were involved with data analysis and interpretation as well as writing the manuscript. All authors read and approved the final manuscript.
